# *Beta vulgaris* Betalains Mitigate Parasitemia and Brain Oxidative Stress Induced by *Plasmodium berghei* in Mice

**DOI:** 10.3390/ph17081064

**Published:** 2024-08-13

**Authors:** Samar A. Khan, Muslimah N. Alsulami, Atif A. Alsehimi, Majed S. Alzahrani, Dina A. Mosule, Haleema H. Albohiri

**Affiliations:** 1 Department of Biology, College of Science, University of Jeddah, Jeddah 21589, Saudi Arabia; sakhan@uj.edu.sa (S.A.K.); mnal-sulami@uj.edu.sa (M.N.A.); damosule@uj.edu.sa (D.A.M.); 2 Department of Medical Laboratory Sciences, Faculty of Applied Medical Sciences, King Abdulaziz University, Jeddah 21589, Saudi Arabia; aaalsehimi@kau.edu.sa (A.A.A.); aaealzahrani@kau.edu.sa (M.S.A.)

**Keywords:** *Plasmodium berghei*, betalains, antioxidants, interleukins, serotonin, epinephrine

## Abstract

Although many drugs have been discovered to treat malaria infection, many of them face resistance from the host’s body with long-term use. Therefore, this study aimed to evaluate the activity of betalains (from *Beta vulgaris*) and chloroquine (a reference drug) against brain oxidative stress induced by *Plasmodium berghei* in male mice. Two protocols were applied in this study: the therapeutic and prophylactic protocols. The results of the therapeutic protocol revealed a significant decrease in the level of parasitemia caused by *P. berghei*. Additionally, the histopathological changes in various brain regions were markedly improved after treatment with betalains. Regarding the prophylactic protocol, betalains were able to protect the brain tissues from oxidative stress, inflammation, and disrupted neurotransmitters expected to occur as a result of infection by *P. berghei*. This was demonstrated by modulating the activities of brain antioxidants (SOD and GSH), inflammatory cytokines (IL-6, IL-10, IL-12, TNF-α, and INF-γ), and neurotransmitters (serotonin, epinephrine, and norepinephrine). This study has proven that using betalains as a treatment or as a preventive has a vital and effective role in confronting the brain histopathological, oxidative stress, and inflammatory changes induced by *P. berghei* infection.

## 1. Introduction

Malaria continues to be one of the most serious global vector-borne infections and a major public health concern [1,2]. Five Plasmodium species—*Plasmodium falciparum*, *P. vivax*, *P. ovale*, *P. malariae*, and *P. knowlesi*—are known to infect humans and constitute the primary pathogen. Of these, *P. vivax* and *P. falciparum* are known to cause the most fatal infections [2,3]. Likewise, *P. knowlesi* is known to cause zoonotic malaria in humans and to infect macaque monkeys [4,5]. The World Health Organization (WHO) reported that malaria is a serious public health issue in 97 countries, accounting for around 219 million new cases annually [2]. In 2020, an estimated 627,000 individuals will be dead from malaria, an increase of 69,000 over 2019. Of those deaths, 47,000 were attributed to disruptions caused by the COVID-19 epidemic. Over 90% of all cases and deaths occurred in Africa, whereas children under the age of five accounted for roughly 70% of all deaths worldwide [2].

Cerebral malaria (CM), severe anemia, acidosis, hypoglycemia, and unexpected renal failure are the most serious side effects of severe malaria [6]. Numerous clinical findings are related to red blood cells’ (RBCs) cytoadherence and sequestration in various organs, such as the brain, lungs, and placenta. Physical signs include, for instance, splenomegaly, jaundice, and pallor [7]. Cerebral malaria is a fatally complicated manifestation of severe *P. falciparum* malaria with fast-rising fatal neurological syndromes and a high rate of mortality among children from sub-Saharan Africa [8,9]. About 1% of *P. falciparum* infections result in CM, which has a high death rate of 15–25% [2,9], leaving its surviving subjects with acute or even persistent physical impairment and neurological dysfunction after the infection has been treated [10].

The pathophysiology of CM is still not well understood. While a number of theories have been proposed on the pathophysiology of CM, including those involving mechanical blockage of microvessels and the release of large quantities of pro-inflammatory cytokines, they fall short of explaining the course, prognosis, and outcome of the disease [11]. The sequestration of iRBCs inside the brain capillary endothelia, which causes microvascular obstruction, blood loss, tissue hypoxia, blood–brain barrier (BBB) impairment, and ultimately CM, is the basis for the vascular occlusion or sequestration hypothesis [3].

The inflammatory theory is based on *P. falciparum* releasing toxic chemicals that lead to an imbalance in systemic inflammatory responses. These responses are further aggravated by iRBC sequestration and cytoadherence [12]. Reactive oxygen species (ROS) and nitric oxide (NO) are released into the bloodstream as a result of the subsequent rise in the release of pro-inflammatory cytokines by macrophages, including tumor necrosis factor-α (TNF-α), interleukin-B1 (IL-B1), and interleukin-10 (IL-10). These cytokines intensify inflammation and break down the blood–brain barrier (BBB) [12,13], leading to fever and impaired erythropoiesis [14].

Although several pathways have been linked to the etiology of malarial infections, oxidative stress is thought to be a key biological factor since it causes anemia and organ damage [15]. The oxidative stress-induced activation of macrophages during Plasmodium infection is indicative of an association between inflammation and ROS [16] and dendritic cells [16], which results in the secretion of inflammatory cytokines. The role of free radicals in the physiopathogenesis of malaria has been addressed by a number of authors. Mice infected with Plasmodium exhibit a progressive rise in oxidative stress and inflammatory cytokines throughout various organs [17]. Monkeys infected with *P. knowlesi* showed high levels of oxidative stress [18], and mice infected with *P. berghei*, *P. yoelii*, or *P. chabaudi* [19] indicated that oxidative stress is a generalized phenomenon in *Plasmodium* infections.

*P. berghei* is a *plasmodium* species from the genus *Plasmodium*; it causes malaria in rodents. *Plasmodium berghei* infection in mice is a commonly used model of experimental CM [20]. The brain, liver, and lymphoid organs represent only some of the organs that are affected by systemic *P. berghei* infection [21]. Therefore, many studies are conducted on rodent malaria to examine resistance, drug screening, and inflammatory cytokines like TNF-α, IFN-γ, and IL-10 [22,23]. Due to its many similarities to human cerebral malaria, the *P. berghei* ANKA strain of rodents has long been utilized as an animal model to investigate the pathogenesis of cerebral malaria [24,25].

The management of malaria mostly depends on vector control and chemotherapy [26]. Unfortunately, the currently available antimalarial drugs are being undermined by emergence and spread of antimalarial-resistant parasites [27], which limit their clinical application and patient compliance. It has been discovered that malaria is resistant to almost every antimalarial medication now in use, such as amodiaquine, chloroquine, mefloquine, quinine, and sulfadoxine-pyrimethamine [28]. The search began for alternate treatments from medicinal plants as the malaria parasite developed resistance to the antimalarial medications. Since they are more effective, less hazardous, and biodegradable, the search for sustainable alternatives, such as plants or oils as well as biopesticides, is an ongoing concern [29,30,31]. Current research has confirmed the potential properties of complementing with antioxidant compounds from plants to strengthen the host’s immune system and improve antioxidant defenses against *Plasmodium* or indirectly aid in the destruction of malaria [29,32]. 

*Beta vulgaris* spp. plants are herbaceous perennial root vegetables, generally known as ‘beetroot’. Many researchers have examined the important anti-inflammatory, antioxidant, and hepato-protective properties of *B. vulgaris* and its constituents [33,34,35]. In mice infected with *P. berghei*, aqueous beetroot extract was reported to exhibit high antimalarial activity [21]. Previous reports have proven that beetroot is rich in minerals and vitamins like vitamin B, vitamin C, and vitamin K [36,37], and high in nitrate content [38]. Additionally, *B. vulgaris* comprises phytochemicals like phenolic acids, flavonoids, saponins, and betalains [36]. Betalains are water-soluble, nitrogen-containing pigments found in the vacuoles of plant cells [39]. Betalains are further divided into two structural groups of pigment-rich compounds, betacyanin and betaxanthin [40]. 

Numerous reports have proven that betalains have high antioxidant and anti-inflammatory abilities to protect cellular components from oxidative injury in in vitro and in vivo animal models [35,41,42]. In humans, betalains demonstrate free radical scavenging actions with potential health benefits, including boosting the immune system and preventing cancer, cardiovascular disease, and neurological diseases [43]. Therefore, this study aims to evaluate the activity of *Beta vulgaris* betalains in comparison with chloroquine (reference compound) against brain oxidative stress induced by *Plasmodium berghei* in male mice.

## 2. Results 

### 2.1. Therapeutic Protocol (Curative Screening)

#### 2.1.1. The Changes in the Parasitemia Level Percentage and Suppression Level

**Figure 1:**
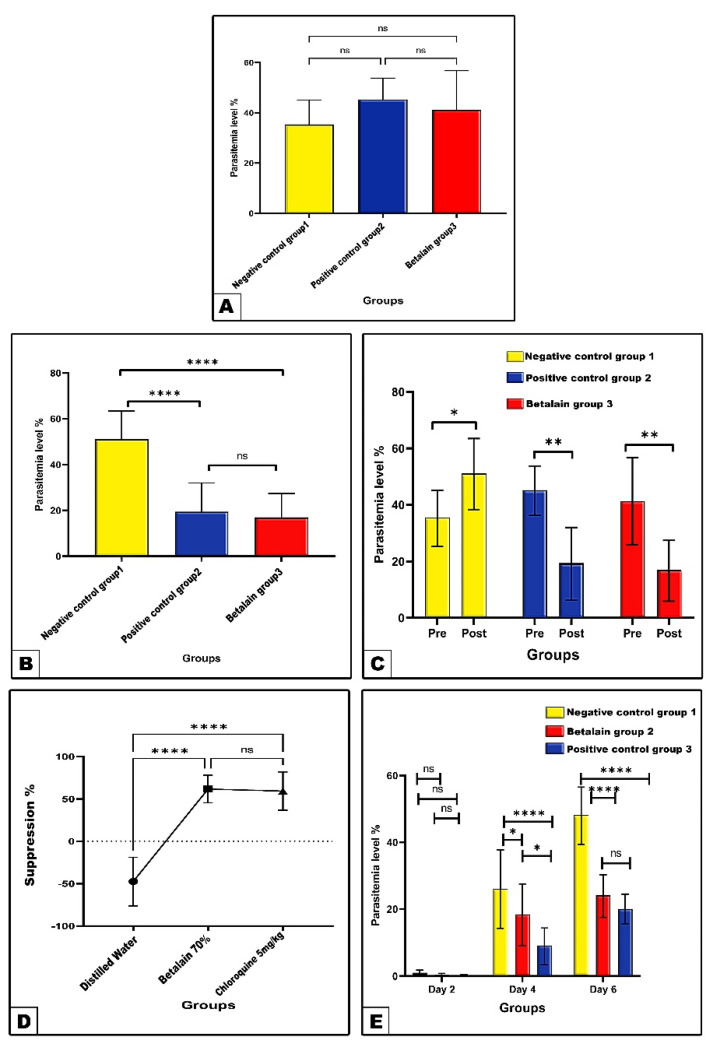
Parasitemia % (Panel (**A**–**C**)) and parasitemia suppression % (Panel (**D**)) before and after treatment with distilled water, chloroquine, and betalains in curative protocol of *P. berghei*-infected mice. Panel (**E**) shows parasitemia % among the different studied experimental groups of mice in prophylactic protocol at 2nd, 4th, and 6th days post-infection with *P. berghei*. Data are expressed as mean ± SD. (ns) non-significance, significance at * *p* < 0.05, ** *p* < 0.01, **** *p* < 0.0001.

The results of the present research revealed in the negative control a parasitemia level of 35.4% at the fourth day post-infection, which significantly increased to 51.2% at the ninth day post-infection with *P. berghei* (Figure 1A and Table 1). Treatment of infected mice with chloroquine (5 mg/kg) or betalains (70 mg/kg) for 5 consecutive days recorded significantly decreased (*p* < 0.01 and *p* ˂ 0.0001) parasitemia % as compared to their corresponding percentages in pre-treatment and the negative control (distilled water) post-treatment, respectively. Meanwhile, treatment with betalains showed a non statistically different decrease (*p* ˃ 0.05) as compared to that of chloroquine in regard to the parasitemia % (Figure 1B,C and Table 1). Accordingly, chloroquine- or betalains-treated groups exhibited significant increased parasitemia suppression% (59.27 and 61.72, respectively) as compared to the negative control group value (−47.46) (Figure 1D and Table 2).

#### 2.1.2. Histopathological Observations of Ions in Brain 

Changes in the cerebral cortex

**Figure 2:**
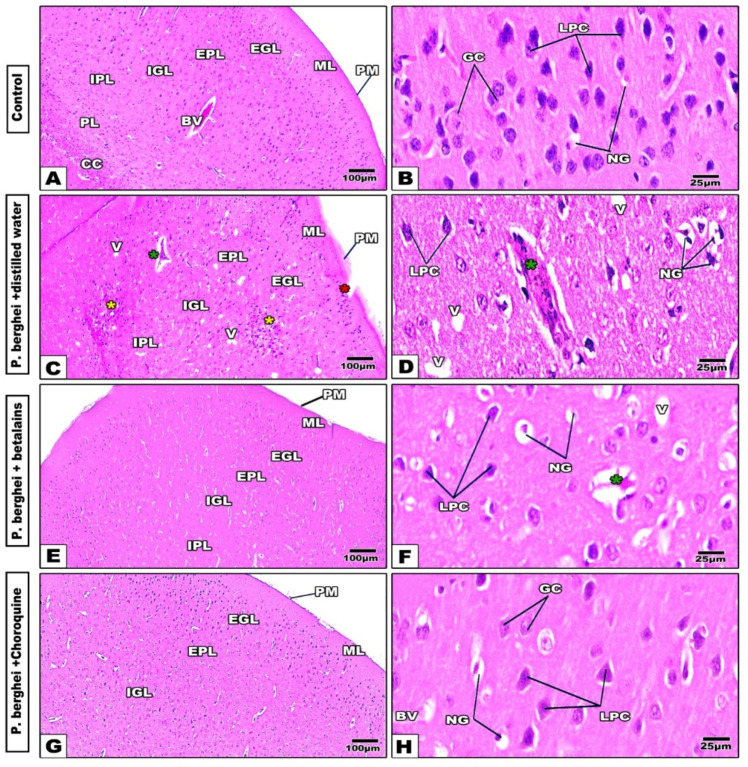
Photomicrographs of histological cerebral cortex sections of mice groups. Normal control group shows well-organized cerebral cortex layers (Panels (**A**,**B**)). The distilled water-treated (negative control) group shows remarkable disrupted molecular layer (red star), cellular infiltration (yellow star), vacuolated cells (V), congested and dilated capillaries (green star), and darkly stained nuclei of neuroglia cells (NG) (Panels (**C**,**D**)). In the betalains (Panels (**E**,**F**)) and chloroquine (Panels (**G**,**H**)) treated groups, cerebral cortex tissue appears more or less similar to the control. (H&E stain). Abbreviations: Pia matter (PM), molecular layer (ML), external granular layer (EGL), external pyramidal layer (EPL), internal granular layer (IGL), internal pyramidal layer (IPL), and polymorphic or multiform layer (PL). Corpus callosum (CC), blood vessel (BV), granular cell (GC), large pyramidal cells (LPC), and neuroglia cells (NG).

The cerebral cortex of control mice (Figure 2A,B) showed normal histological features of the cortical tissue cell layers, molecular layer (ML), external granular layer (EGL), external pyramidal layer (EPL), internal granular layer (IGL), internal pyramidal layer (IPL), and polymorphic or multiform layer (PL). In the infected negative control group, the cerebral layers appeared disorganized with disrupted ML, remarkable cellular infiltration in the EPL and IPL, vacuolated granular cells, darkly stained nuclei of neuroglial cells, and some congested and thickened wall blood capillaries (Figure 2C,D). The betalains-treated group showed histological remarkable amelioration by disappearance of the cellular infiltration, vacuolation, and dilated capillaries (Figure 2E,F). Meanwhile, the chloroquine-treated group showed improvement in the histological appearance of the cortical layers (Figure 2G,H). Hence, both treatments showed relative similarities in restoration of cerebral histopathological signs induced by *P. berghei* infection.

Changes in the cerebellar cortex

**Figure 3:**
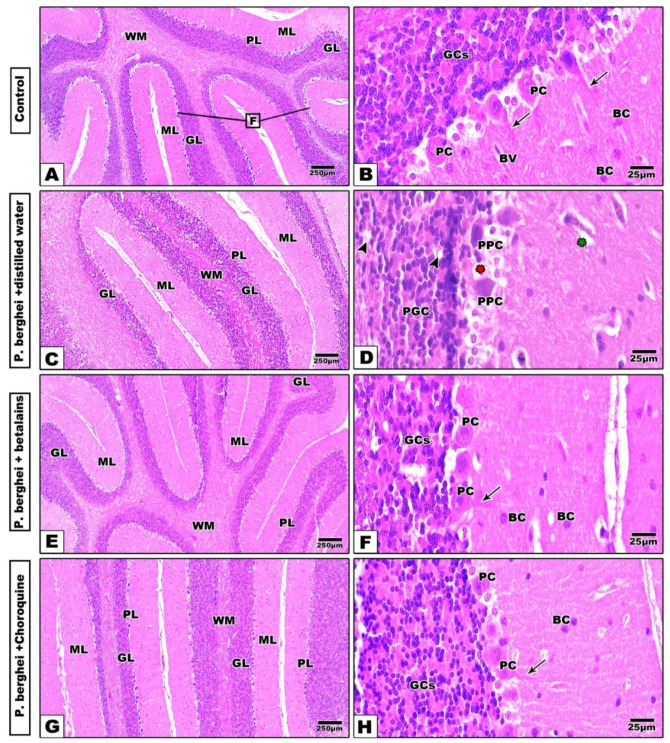
Photomicrographs of histological cerebellar cortex sections of mice groups. Normal control group shows well-organized architecture, cerebellar cortex layers, and folia (Panels (**A**,**B**)). In the distilled water-treated (negative control) group, the cerebellar cortex appears less foliated with dilated capillaries in the ML, lysed (red star) and pyknotic Purkinje cells (PPC), vacuolated and aggregated (arrow head), congested and dilated capillary (green star) and pyknotic granular cells (PGC) (Panels (**C**,**D**). In the betalains (Panels (**E**,**F**)) and chloroquine (Panels (**G**,**H**)) treated groups, the layers and folia of the cerebellar cortex appear more or less similar to the control (H&E stain). Abbreviations: Cerebellar folia (F), molecular layer (ML), Purkinje cell layer (PJ), Purkinje cell (PC), granular layer (GL), granular cells (GCs), and basket cell (BC), blood vessel (BV), white matter (WM), and arrows point to the dendrites of PC.

**Figure 4:**
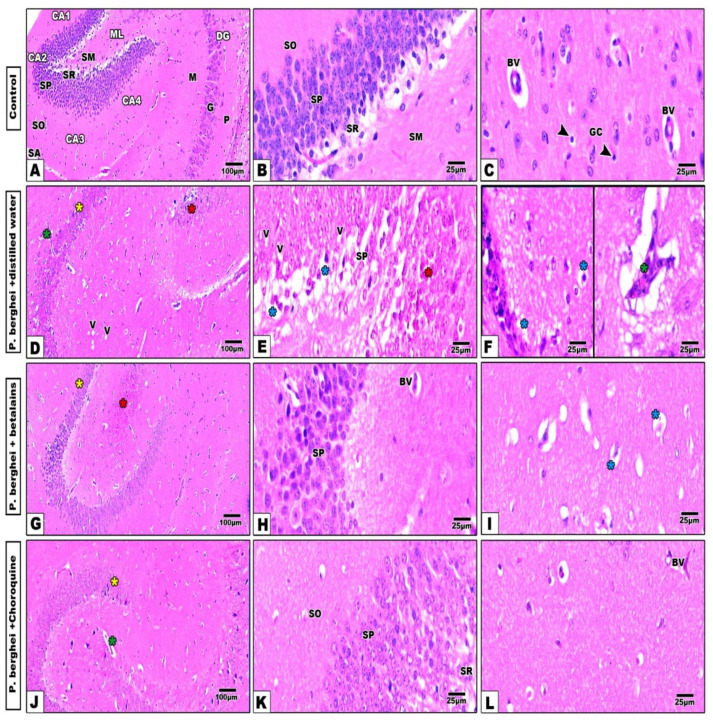
Photomicrographs of hippocampus histological sections of control mice (Panel (**A**–**C**)) shows a well-organized regions architecture of the hippocampus. In infected mice treated with distilled water (Panel (**D**–**F**)), the hippocampus cell layers show several deformities especially in the proper (yellow asterisk) and dentate gyrus zones (red asterisk) with prominent vacuolation (V) and pyknosis (blue asterisks) in their cells and dilated capillaries (green asterisk). Treatment of infected mice with betalains (Panel (**G**–**I**)) or chloroquine (Panel (**J**–**L**)) display remarkable improvement in the histological architecture of hippocampus compartments in spite of little deformities still present especially in the cornu ammonis and dentate gyrus zones (H&E stain). Abbreviations: Blood vessel (BV), Cornu ammonis (CA), dentate gyrus (DG), molecular layer (ML), stratum alveolus (SA), stratum oriens (SO), stratum pyramidale (SP), stratum radiatum (SR), stratum molecular (SM), polymorph layer (P), granular layer (G), molecular layer (M), glial cells (GC) (arrow head).

Histologically, the cerebellar cortex from the control mice appeared with normal architectural patterns, whereas the cerebellar folia and layers are well-organized. Each folium is made of an outer molecular layer (ML), middle Purkinje cell layer (PL), and an inner granular cell layer (GL). The ML contains little basket cells and the dendrites of Purkinje cells. The GL appeared rich with a high density of tightly packed small granular cells. The PL consisted of one raw layer of large and pear-shaped neurons with prominent centrally located nuclei and basophilic cytoplasm (Figure 3A,B). Sections of cerebellar cortex from infected mice treated with distilled water showed remarkable histopathological changes, including a less foliated cerebellar cortex with dilated and congested capillaries in the ML, lysed and pyknotic Purkinje cells, and vacuolated, aggregated, and pyknotic granular cells (Figure 4C,D). Meanwhile, treatment with betalains (Figure 3E,F) or chloroquine (Figure 3G,H) successfully restored the altered histopathological signs caused by *P. berghei* through the apparent well-organized architecture of cerebellar folia and their layers, while some vacuolated cells still appeared in the sections. 

Changes in the hippocampus 

In control mice, histological sections of the hippocampus showed the usual structure of its distinct regions that were represented by the hippocampus proper, dentate gyrus, and subiculum. The hippocampus proper is formed from the C -shaped cornu ammonis (CA), which differentiates into four zones; CA1, CA2, CA3, and CA4. CA1 and CA2 contain small pyramidal cells, while CA3 and CA4 are rich with large pyramidal cells and are continued as subiculum. Moreover, the four zones of the hippocampal proper exhibit five strata: stratum alveolus (SA), stratum oriens (SO), stratum pyramidale (SP), stratum radiatum (SR), and stratum molecular (SM). The SP layer contains large-sized pyramidal neurons. The dentate gyrus (DG) is seen surrounding CA4 by its upper and lower extensions and is well differentiated into three distinct layers: the outer polymorph (P), middle granular (G), and inner molecular (M) cell layers. The molecular layer (ML) appears at the concavity of the CA and of the DG. The granular cells of the DG appeared compact with darkly stained nuclei as well as pyramidal cells also seen. Furthermore, the molecular (M) and polymorphic (P) layers among neuronal processes, glial cells (GC), and blood capillaries (BC) are visible (Figure 4A–C).

In *P. berghei*-infected mice treated with distilled water, the hippocampus cell layers showed several deformities especially in the proper and dentate gyrus zones, with prominent multiple cytoplasmic vacuolation all over their granular and pyramidal cells. Additionally, remarkable aggregation as well as apoptotic and pyknotic cells were noticed in the four zones of the hippocampus proper (Figure 4D–F). On the other hand, treatment of *P. berghei*-infected mice with betalains (Figure 4G–I) or chloroquine (Figure 4J–L) revealed remarkable improvement in the histological architecture of hippocampal compartments in spite of some little deformities still appearing especially in the cornu ammonis regions.

### 2.2. Protective Protocol (Prophylactic Screenings)

#### 2.2.1. The Changes in Parasitemia Level 

In this protocol, the experimental groups were primarily treated with the target substances for four consecutive days, then inoculated with *P. berghei* on the fifth day. The parasitemia at days 4 and 6, as compared to the corresponding negative control (distilled water), values were significantly decreased in the betalains protective group recordings (18.33% and 24.16%), with estimated inhibition % of 29.50% and 49.82%, respectively. Likewise, there were significant decreases in the chloroquine-treated protective group recording parasitemia (9.00% and 20.00%), with estimated inhibition % of 65.38.50 and 58.47%, respectively. Only the chloroquine group showed significantly (*p* < 0.05) decreased parasitemia % at day 4 compared with the betalains group, (Figure 1E and Table 3).

#### 2.2.2. The Changes in Hematological Parameters

**Figure 5:**
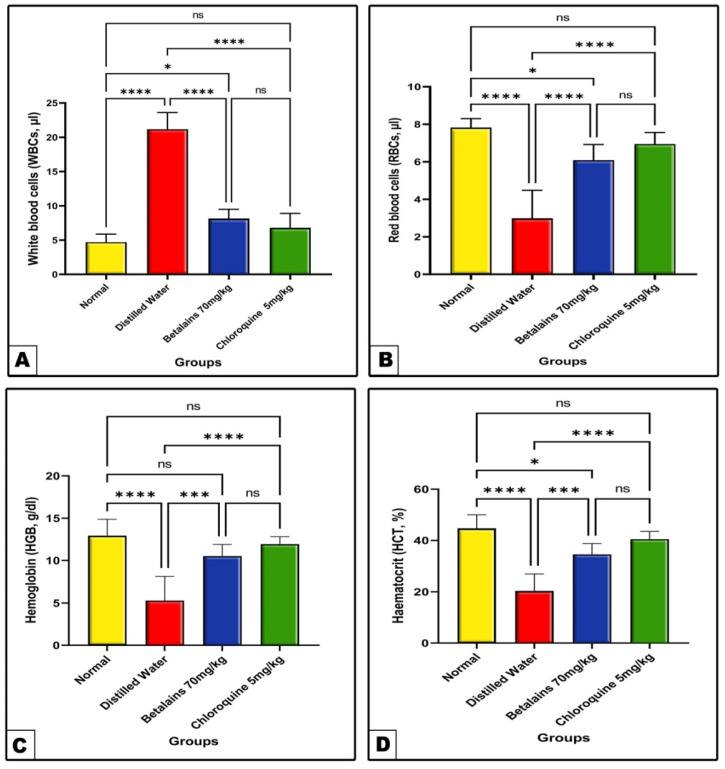
The mean level of total WBCs (Panel: (**A**)) and, RBCs (Panel: (**B**)), HGB (Panel: (**C**)) and HCT (Panel: (**D**)) of the normal control and the three prophylactic groups of mice. Data are expressed as mean ± SD. (ns) non-significance, significance at * *p* < 0.05, *** *p* < 0.001, **** *p* < 0.0001.

As shown in Figure 5, the complete blood count (CBC) analysis from the negative control (distilled water) infected mice group revealed a significant (*p* < 0.0001) increase in the total WBCs count, while the RBC count and hematocrit and hemoglobin levels were significantly decreased (*p* < 0.0001) as compared to the corresponding normal control values. Meanwhile, in prophylaxis of the betalains or chloroquine groups, data showed remarkable amelioration as compared with the negative group values. In the meantime, the chloroquine prophylactic group showed prominent but not statistically different recovery of CBC compared to the betalains group. 

#### 2.2.3. The Changes in Biochemical Assays in Brain Tissues

Changes in antioxidant markers (NO, SOD, and GSH) and MDA

**Figure 6:**
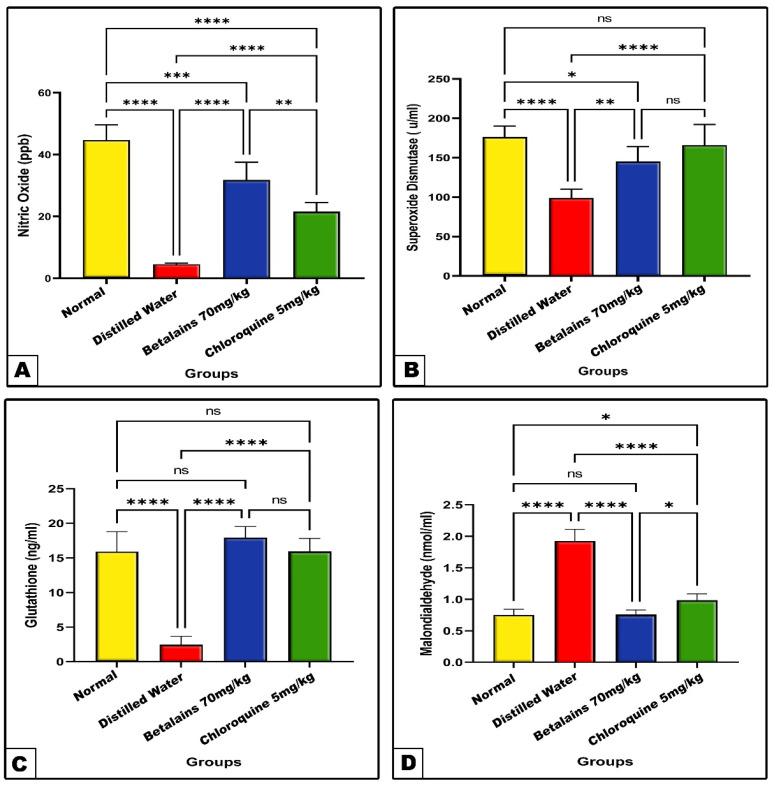
The mean level of nitric oxide (Panel: (**A**)), superoxide dismutase (Panel: (**B**)), glutathione (Panel: (**C**)) and malondialdehyde (Panel: (**D**)) of the normal control and the three prophylactic groups of mice. Data are expressed as mean ± SD. (ns) non-significance, significance at * *p* < 0.05, ** *p* < 0.01, *** *p* < 0.001, **** *p* < 0.0001.

In the *P. berghei*-infected group previously treated with distilled water for four consecutive days showed a significant decrease (*p* < 0.0001) in the brain tissue NO and GSH contents and SOD enzyme activity, in line with the significant increase (*p* < 0.0001) in MDA level as compared with normal control group values. On the other hand, the NO and GSH levels and SOD activity were significantly increased and the MDA level significantly declined in betalains or chloroquine protected groups as compared with the negative control (Figure 6). Comparatively, the prophylactic role of betalains appeared more prominent than that of chloroquine against deleterious changes in the brain antioxidant markers induced by *P. berghei*. 

Changes in inflammatory cytokines’ markers 

**Figure 7:**
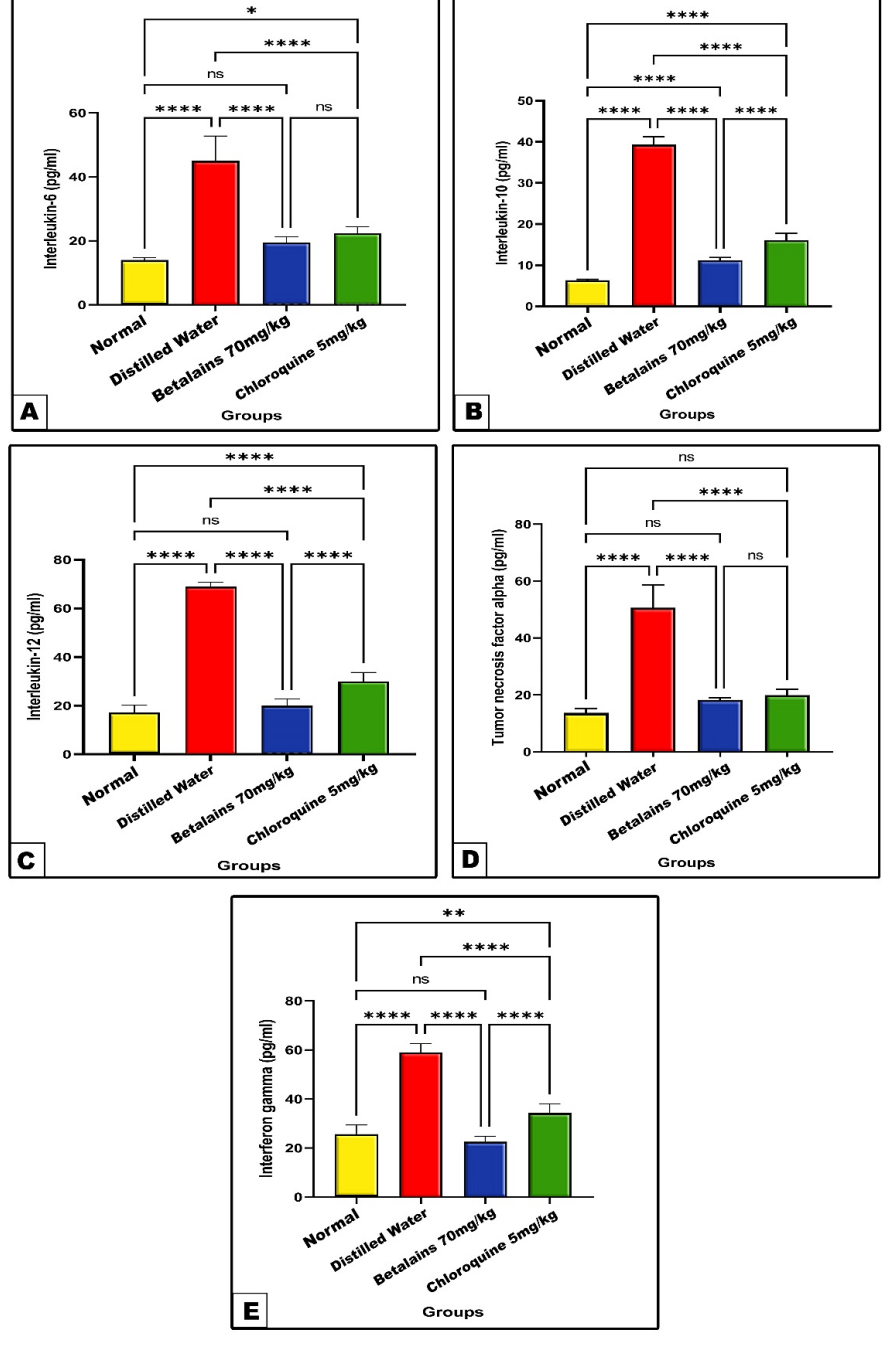
The mean level of of interleukins, IL-6 (Panel: (**A**)), IL-10 (Panel: (**B**)), IL-12 (Panel: (**C**)), TNF-α (Panel: (**D**)), and IF-γ (Panel: (**E**)) of the normal control and the three prophylactic groups of mice. Data are expressed as mean ± SD. (ns) non-significance, significance at * *p* < 0.05, ** *p* < 0.01, **** *p* < 0.0001.

The negative control group showed significant increase (*p* < 0.0001) in the levels of brain tissue-tested inflammatory cytokines IL-6, IL-10, IL-12, TNF-α, and INF-γ as compared with the normal control values. On the contrary, the activities of these inflammatory markers were significantly decreased (*p* < 0.0001) in the betalains and chloroquine groups as compared with the negative control values. Meanwhile, the betalains protective group exhibited a restoring to normal inflammatory cytokines range as compared to normal control values with the exception of IL-10, which significantly increased (*p* < 0.0001) compared to the normal value (Figure 7). 

Changes in brain neurotransmitters (Epinephrine, Norepinephrine, and Serotonin).

**Figure 8:**
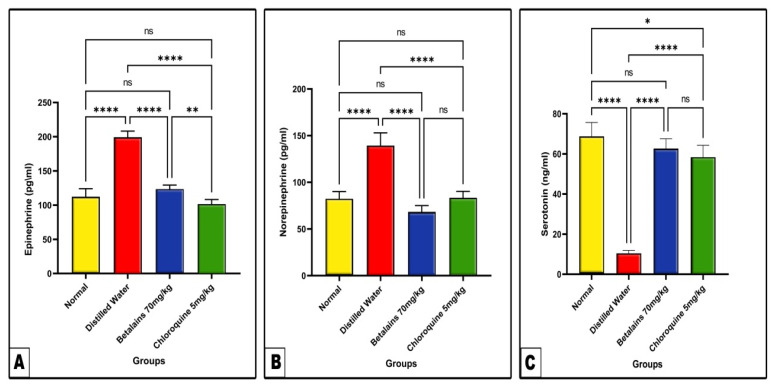
The mean levels of neurotransmitters epinephrine (Panel: (**A**)), norepinephrine (Panel: (**B**)) and serotonin (Panel: (**C**)) in the brain tissues of the control and the three prophylactic groups of mice. Data are expressed as mean ± SD. (ns) non-significance, significance at * *p* < 0.05, ** *p* < 0.01, **** *p* < 0.0001.

As shown in Figure 8, the levels of brain tissue epinephrine and norepinephrine appeared significantly elevated (*p* < 0.0001) in the negative control group, while the level of serotonin was significantly decreased (*p* < 0.0001) as compared with the normal control values. In the betalains group, the levels of epinephrine, norepinephrine, and serotonin did not show statistically significant differences as compared to normal control values. Meanwhile, chloroquine prophylactic treatment showed restoration of epinephrine and norepinephrine levels to normal levels, while the level of serotonin was still significantly higher (*p* ˂ 0.05) than the normal control value.

## 3. Discussion 

The search for an adequate antimalarial medication has surged; however, the emergence and propagation of antimalarial-resistant parasites has slowed down this attempt [27]. One class of naturally occurring pigments found in around 17 distinct plant families are called betalains, which are considered bioactive chemical compounds [44]. Betalains are currently being used as pharmaceutical therapeutic agents [45,46]. Even at relatively high concentrations, betalains have been shown to be nontoxic and safe for administration for developing cell cultures [39,47]. Accordingly, the present study aimed to discriminate the therapeutic and prophylactic impacts of betalain compounds (from *Beta vulgaris*) in parallel with chloroquine (standard antimalarial drug) against brain oxidative stress induced by *P. berghei* in male mice. 

The obtained results demonstrated that the treatment of *P. berghei*-infected mice with distilled water for five consecutive days following infection for four days significantly increased the parasitemia percentage level. However, on corresponding treatment of *P. berghei*-infected mice with betalains or chloroquine, the level of parasitemia significantly decreased as compared to the negative control. This indicates that betalains are effective against malaria, but they are not significantly more effective than the usual treatment with chloroquine. The data concerned with the therapeutic activity of betalains in this study are in line with previous reports [39,48] that found pronounced antimicrobial and antimalarial effects of betalains. Hilou et al. found that betalains from *Amaranthus spinosus* and *Boerhaavia erecta* extracts can suppress parasitemia in *P. berghei*-infected mice [49]. This is in line with the present study, as previously mentioned betalains constitute betacyanins and betaxanthins pigments, and betacyanins comprise 75–95% betalains with the rest as betaxanthins. In addition, betanin, isobetanin, and neobetanin are the main betacyanins as previously reviewed [50]. In regard to betanin (betanidin 5-O-glucoside; CAS 37279-84-8), the major constituent of betalains contains quaternary nitrogen in its structures, which is a chemical group that is the most abundant betalain in beetroot and is well known to inhibit *Plasmodium* growth by blocking the parasite’s choline intracellular transport necessary for the biosynthesis of the essential molecules for the *Plasmodium*, which are the phosphatidylcholines [39,51]. Chang et al. added that betacyanins, which are a main constituent of betalains, can attenuate dengue virus type 2 [52]. Additional research demonstrated the antimicrobial activity of betalains by preventing the growth of bacteria within the cells [53,54]. The ability of betacyanins, a key component of betalains that chelate inner cations (Ca^2+^, Fe^2+^, and Mg^2+^) and prevent the intracellular transport of choline in parasites, may be one the mechanisms by which betalains could reduce parasitemia [49,55]. The most widely used mouse models for researching malaria pathophysiology in various organs are those infected with *P. berghei* ANKA [56,57]. The curative test, therefore, was designed to look into the histological alterations in the brain tissues of the groups that we studied.

In our current research, inoculation of male mice with *P. berghei*-infected RBCs showed remarkable histopathological signs in the different brain regions including the cerebral cortex, cerebellar cortex, and hippocampus. On the other hand, post-treatment of *P. berghei*-infected mice with chloroquine or betalains, most of the histopathological signs induced by *P. berghei* apparently disappeared. With regard to the cerebral cortex, *P. berghei*-infected mice treated with distilled water showed disorganized cerebral cortex layers with disrupted ML, cellular infiltration in the EPL and IPL, vacuolated granular cells, darkly stained nuclei of neuroglial cells, as well as some blood capillaries appearing congested with obvious thickened walls. Our histopathological results on the cerebral cortex are similar to previous histological characteristics of the cerebral malaria model in mice induced by *P. falciparum* infection [58,59].

Regarding the histopathological changes caused by *P. berghei* infection on the cerebellar cortex in this study, they included a less foliated cerebellar cortex with dilated and congested capillaries in the ML, lysed and pyknotic Purkinje cells, and vacuolated, aggregated, and pyknotic granular cells. The obtained results are in agreement with those of previous studies [60,61,62] where pronounced histopathological features were found in the cerebellar cortex in *P. berghei ANKA*-infected mice as a malarian model.

In addition to the induction of histopathological aspects in the cerebral and cerebellar cortex of *P. berghei*-infected animals treated with distilled water, the hippocampal cell layers showed signs of disarray, particularly in the proper and dentate gyrus zones. Also, some granular and pyramidal cells appeared vacuolated while other cells appeared pyknotic or apoptotic in the four zones of the hippocampus proper. Consistent with our findings, Bedri et al. observed significant hemorrhage, inflammation, and apoptosis in an experimental cerebral malaria model [63].

Previous research has demonstrated that exposure to malaria infection may result in increased secretion and release of pro-inflammatory cytokines, which can cause endothelial activation, disrupt the blood–brain barrier, and trigger neurodegenerative events that cause neurological and cognitive dysfunction in the brain regions in which histopathological changes have been observed [9,64]. Additional findings have demonstrated that pRBCs may provoke inflammation in the brain by upregulating the production of TNF-α, CD54, and inflammatory cytokines [63,65].

Data from the current study that address the therapeutic action of chloroquine against the histopathological features of the brain caused by *P. berghei* are consistent with those from other studies that investigated chloroquine as an antimalarial drug [61,62]. According to Zhang et al., chloroquine, as a prototype anti-malaria drug, showed anti-inflammatory effects, demonstrated a high degree of penetration into the brain’s membranes, and has been shown to play an important role in suppressing the DNA activity responsible for brain injury [66]. 

Regarding the ameliorative role of betalains against *P. berghei*-induced histopathological signs in brain regions could be explained by the strong antioxidant properties of betalains that are triggered by the presence of betacyanins that include betanidin and betanin [67,68]. According to Moreno-Ley et al., betalains have a significant and useful role in protecting brain tissues from the damaging effects of malaria [69]. A related study also found that betalains, which are isolated from beetroot, had a strong ameliorative impact on renal tissue from oxidative stress caused by gentamycin [70].

Betalains have been shown to have a possible therapeutic effect against *P. berghei*-induced brain tissue damage. This has prompted additional research to investigate the preventive role of betalains against malaria infection by estimating certain biochemical markers associated with brain pathophysiology. Data from the prophylactic screening indicated that 4 and 6 days post-parasite inoculation, the percentage level of parasitemia was much lower in the groups that had received betalains or chloroquine pretreatment than in the negative control. The results showed that the preventive parasitemia activity of betalains was predicted, and the results showed no significant difference in the effect of betalains when compared to the reference drug. The obtained results were in agreement with those published by Albohiri et al., who found a significant decrease in the parasitemia percentage level using *Beta vulgaris* aqueous extract in *P. berghei*-infected mice [21]. Additionally, various investigations have already assessed models of betalains used to mitigate plasmodium activity [39,49,51]. A related study by Wondafrash et al. showed the potent preventive effect of *Cordia Africana* leaf extract against parasite load in mice infected with *P. berghei*. [71] The authors attributed the antimalarial activity of this extract to the presence of the total flavonoids and total phenols. Our obtained results revealed that the prophylactic role of betalains against parasitemia level was less prominent than its curative role. This may be attributed to the rapid hepatic clearance or metabolism and the higher bioavailability of the active component responsible for antimalarial activities, as previously reported by Alehegn et al. [72].

Malaria is thought to be indicated by hematological abnormalities, and statistical studies have demonstrated that a number of these hematological values may raise the clinical suspicion of malaria. Numerous hematological changes, including leukocytosis, thrombocytopenia, and steadily rising anemia, have been associated with malaria cases [73]. According to our findings, the group that received distilled water pretreatment had significantly lower RBC counts, and higher leukocyte counts than the control group, together with significantly lower mean values of hemoglobin and hematocrit. This demonstrates that leukocytosis-induced inflammation and a decrease in hemoglobin and RBC counts are the results of malaria-induced anemia. The results obtained are consistent with the earlier reports [73,74] which recorded remarkable anemia and inflammation in *P. berghei* infected models**.** Moreover, leukocytosis was discovered to be associated with malaria infection [75], which is consistent with our results. RBCs are one of *Plasmodium’s* main targets, which accounts for the majority of the alterations in hematological parameters associated with malaria infection [76]. According to Onohuean et al., hemoglobin degradation and RBC rupture followed by the parasite are the main causes of the hemoglobin level drop associated with malaria infection [77].

Oxidative stress is brought on by the *Plasmodium* parasite infection and is manifested by a redox imbalance in the host as a result of increased production of reactive oxygen and nitrogen species (ROS/RNS). The parasite destroys hemoglobin to obtain amino acids for its own nutrition, and the host’s immune system also produces ROS/RNS inside the phagocytes, resulting in the production of these oxidizing radicals [78]. Moreover, malaria induces a rise in oxidative stress, which damages tissues and organs, including the brain and lungs. Depending on the lesions in the brain parenchyma, this redox imbalance can cause both physical and cognitive problems and has been linked to the more serious forms involving cerebral malaria [79]. In our research, *P. berghei*-infected mice revealed a significant increase in the activity of brain tissue oxidative stress markers, MDA (lipid peroxidation), and a remarkable decrease in the activities of SOD and GSH. This supports that a malarial infection causes oxidative stress in the brain. Our obtained results are in parallel with the findings of Al-Shaebi et al. and Gomes et al., who recorded a significant increase in MDA activity with a remarkable decrease in brain tissue CAT and GSH antioxidants in *P. berghei*-infected mice [32,80]. Since GSH is a crucial substrate for *Plasmodium* replication in its vertebrate host, the study’s reported decrease in GSH may be explained by this consumption [81].

Increasing pro-inflammatory cytokines like TNF-α and IF-γ, as well as certain interleukins like IL-6, IL-10, and IL-12, is probably a key to the etiology of cerebral malaria [82]. Our obtained data showed a remarkable increase in the levels of inflammatory cytokines in the brain tissues of *P. berghei*-infected mice pre-treated with distilled water. It has been observed that clinical or severe malaria is associated with increased levels of pro-inflammatory cytokines, including IL-1β, IL-6, IL-8, IL-10, IL-12, IL-13, IL-31, IL-33, and TNF-α [83,84]. Moreover, pro-inflammatory cytokine overexpression induces brain endothelial cells to generate certain adhesion molecules, which in turn causes platelets, leukocytes, and platelets to adhere to the brain endothelium and trigger damage to brain tissue. It has been observed that TNF-α, IL-31, and IL-33 are associated with clinical or severe malaria [85,86]. Furthermore, it has been established that IL-12 influences TNF-α and IF-γ expression. In the first phase of infection, dendritic cells or macrophages may produce IF-γ at an appropriate increased level in response to IL-12 [87,88], which remains a crucial component of pro-inflammatory responses. Simultaneously, the IF-γ response has the ability to upregulate TNF-α, indicating that it is a major mediator of malaria pathogenesis [89]. Evidence has been found to suggest that high plasma levels of TNF-α are related to malaria infection [90]. 

The central nervous system (CNS) produces IL-10, which reduces the clinical symptoms of meningitis, stroke, multiple sclerosis, Alzheimer’s disease, and infection-related behavioral abnormalities. The majority of neurological conditions are accompanied by raised levels of IL-10, which protects brain neurons and all glial cells from damage by inhibiting the actions of pro-apoptotic cytokines while promoting the expression of cell survival signals [91]. Immune cells produce IL-6 when infected with malaria parasites, and this has been associated with the immunopathogenesis of malaria [92]. Additionally, the malarial infection has been associated with ROS formation that triggers the release of TNF-α and IL-6 [77].

In the current study, the levels of brain inflammatory cytokines markedly declined to near-normal values with the prophylactic use of betalains against *P. berghei*-infected mice. Betalains were found to regulate inflammation processes such as NF-κB regulation, as well as attenuate pro-inflammatory cytokines and stimulate anti-inflammatory mediators [93]. It has been documented that betalains reduced inflammation and restored function to several organs such as the lungs and airways [94], heart [95,96], gut [97], liver [95], kidney [98], and reproductive organs [99]. Shunan et al. investigated the application of betalains to mitigate the inflammation and oxidative stress caused by aluminum chloride in brain tissues [100]. The findings showed a notable reduction in MDA maintenance of antioxidant enzymes like SOD, GSH, and CAT. In addition, betalains alleviated the expressions of pro-inflammatory cytokines—IL-6, IL-1β, COX-2, iNOS, TNF-α, and NF-κB—via mRNA expression reversion. Furthermore, it has been revealed that betanin, the main ingredient of betalains, inhibits the in vitro lipopolysaccharide-induced inflammation of microglial cells by reducing the generation of ROS, RNS, TNF-α, IL-1β, and IL-6 [101]. According to Imamura et al., betalains and iso-betalains protect mitochondria by reducing the production of ROS, which in turn reduces inflammation in the brain caused by amyloid-β [102]. Betanin was indicated as a COX-2 inhibitor [103]. Betanin anti-inflammatory effects, including TNF-α and IL-β reduction and increased IL-10, were also reported by Martinez et al. [104]. This suggests that betalains and their derivatives contribute to organ protection in disease conditions via the regulation of antioxidant and anti-inflammatory processes, which are in agreement with our obtained results.

Neurotransmitters are essential molecules of central and peripheral nervous systems. These molecules are crucial for signaling, enabling nerve cells, or neurons, to effectively transmit information both chemically and electrically [105]. The impairment of some neurotransmitters was noticed in various parasitic infections such as *Toxoplasma* [106], *Schistosoma mansoni* [107], *Toxocara canis*, and *Trichinella spiralis* [108]. According to Clark et al., neurotransmitter activity impairment has been linked to the etiology of CM [109]. In the current study, we observed an increase in the activities of some brain neurotransmitters like epinephrine and norepinephrine, while a decrease in serotonin was noted among *P. berghei*-infected mice pre-treated with distilled water. Previous reports have declared that the increase in the activities of epinephrine and norepinephrine during malaria infection may help the host overcome the risk of fever [80,110]. Also, Roy et al. reported a remarkable decrease in level of brain tissue serotonin in *P. berghei*-infected mice [111]. Additional research has demonstrated that one of the signs of malaria is neuro-degeneration of brain tissues, which results in an imbalance in neurotransmitter levels [101,112].

In our current study, the pretreatment of *P. berghei*-infected mice with betalains successfully attenuated the deterioration of brain neurotransmitters caused by malaria to nearly normal values as the control. This suggests the prophylactic role of betalains against neurodegenerative effects induced by malaria. Studies have shown that betalains function as neuroprotective compounds by mitigating the oxidative stress and inflammation brought on by neurotoxicants [100], amyloid-β aggregation [102], mitochondrial dysfunction [113], and brain microglia activation [101], thus preventing neurodegeneration [114]. According to Thong-asa et al., betanin can improve brain antioxidants, which may have a neuroprotective impact against trimethyltin-induced neurodegeneration in rats [115]. The researchers also evaluated the oxidation levels in brain tissue and found that there was a notable decrease in MDA levels and an increase in catalase and SOD activity. 

Collectively, the results of the present study are in line with the previously reviewed mechanism of betalains from previous studies that suggested betalains may bind with heme and inhibit hemozoin formation in *Plasmodium* parasite in a similar action to chloroquine that interferes with the degradation of erythrocyte hemoglobin to prevent parasite growth by the accumulation of its toxic material hematin. This is in line with the antioxidant activity of betalains, which at low concentrations has been demonstrated to inhibit lipid peroxidation and heme decomposition [44]. Also, this is in line with betalains’ previously predicted antioxidant and anti-inflammatory activities [44,101]. 

## 4. Materials and Methods 

### 4.1. Experimental Mice

In the current study, 48 male Swiss albino mice, weighing between 19 and 26 g and aged between 6 and 8 weeks, were obtained from the Experimental Animal Unit at the King Fahad Medical Research Centre, King Abdulaziz University in Jeddah, Saudi Arabia. The mice were kept in individually ventilated cages (IVC) constructed from protective plastic to keep the concentrations of ammonia and CO_2_ low and prevent the spread of diseases. The mice were kept in a lab environment with controlled humidity (65%), room temperature (20 ± 2), and artificial lighting with a 12-h cycle of light and dark. The mice were provided with free access to commercial chow and were fed a standard diet ad libitum. 

*Plasmodium berghei* inoculation

The *Plasmodium berghei* “ANKA Strain”, which is prone to chloroquine, was initially obtained from the Institute of Immunology and Infection Research located in the European Malaria Reagent Repository in Edinburgh. Liquid nitrogen was used to preserve the infected blood for use in donor mice. Four donor mice received intra-peritoneal injections of 80 μL, 100 μL, 130 μL, and 170 μL of cryopreserved *Plasmodium berghei* parasitized red blood cells (pRBCs) after they had thawed at room temperature. The air-dried blood films were fixed with 100% methanol, stained with 10% Giemsa stain solution, and inspected under an high-power-objective microscope in order to confirm the infection [116]. 

According to Rahayu et al., the number of parasitized erythrocytes within at least 1000 RBCs was assessed by counting ten fields of around 100 erythrocytes each, and the parasitemia in mice was then calculated using the following equation: (Number of parasitized RBCs/Total number of tested RBCs) × 100 [117]. Before being bled for *Plasmodium* infection, donor mice had parasitemia levels between 2 and 15% [118]. Following the confirmation of the parasitemia level, 0.2 mL of a solution made from 10 µL of infected mouse blood and 5 mL of phosphate-buffered saline (PBS) was intraperitoneally administered into a new mouse as part of the mechanical passage procedure.

The experimental mice were injected intraperitoneally with a 0.2 mL suspension of 1 × 10^6^ parasitized RBCs of the *P. berghei* ANKA strain, in accordance with the Reece Lab Protocols from The European Malaria Reagent Repository. The inoculated mice served as both a source of experimental mice and as passage mice for the course of the study, and were supplied with food and water in cages intended for experimental animals. All procedures were approved by the Unit of Biomedical Ethics Research Committee at King Abdulaziz University, Saudi Arabia, with animal study approval (reference no. 657-19).

### 4.2. Chemical Compounds Used

Betalains derived from *Beta vulgaris* was purchased from Shaanxi Baichuan Kangze Biological Technology Company (Xi’an XABC Biotech Co., Ltd. Xi’an, China), CAS No.: 37279-84-8 [39,119]. With regard to betalains used in the present study under CAS No.: 37279-84-8 according to the Open Chemistry Database (PubChem) at the National Institutes of Health (NIH), betalains chemicals are compounds derived from TYROSINE via betalamic acid, including betaxanthins and betacyanins. They are found in the Caryophyllales order of plants that *Beta vulgaris* belongs to and with the IUPAC name (2R)-1-[(2E)-2-(2,6-dicarboxy-2,3-dihydro-1H-pyridin-4-ylidene) ethylidene]-6-hydroxy-5-[(2R,3R,4S,5S,6R)-3,4,5-trihydroxy-6-(hydroxymethyl)oxan-2-yl]oxy-2,3-dihydroindol-1-ium-2-carboxylate; https://pubchem.ncbi.nlm.nih.gov/compound/56841626 (accessed on 12 August 2024).

Chloroquine diphosphate was purchased from Shanghai Huirui Chemical Technology Company’s preferred medication (Hui Chem Co., Ltd., Shanghai, China) CAS No.: 50-63-5 and Bach No. HR2019060801.

### 4.3. Antimalarial Activity 

In this study, we performed two screening protocols to evaluate the antimalarial activity; the primary protocol is therapeutic, and the secondary is the prophylactic protocol according to Fidock et al., with a few modifications [120]. 

#### 4.3.1. Therapeutic Protocol (Curative Screening) 

Twenty-four mice were utilized in this experiment; eighteen of the animals received an intraperitoneal injection of a standard inoculum consisting of 10^6^ *Plasmodium berghei*-infected RBCs in 2.97 mL of PBS with an 18% parasitemia level. Following 96 h, on the fifth day of the experiment, the mice were divided into four groups, with six mice per group, based on the confirmation of the level of parasitemia. 

Group 1: Negative control group; mice were infected with 10^6^ *Plasmodium berghei*-infected RBCs followed by oral administration of distilled water. 

Group 2: Positive control group; mice were infected with 10^6^ *Plasmodium berghei*-infected RBCs followed by oral administration of 5 mg/kg of chloroquine.

Group 3: Mice were infected with 10^6^ *Plasmodium berghei*-infected RBCs followed by oral administration of 70 mg/kg of betalains. 

Group 4: Normal NOT infected mice as control.

Following the fourth day of infection, all tested substances were administered to groups G1, G2, and G3 for five consecutive days at a single dose. Blood smears for each mouse in each group were collected at the end of the experiment (day 9) and examined under a microscope to track any differences in parasitemia between the groups. 

Determination of parasitemia percentage and suppression effect

According to Rahayu et al., the number of parasitized RBCs within at least 1000 RBCs was assessed by counting ten fields of around 100 erythrocytes each [117], and the parasitemia in mice was then calculated using the following equation: (Number of pRBCs/Total number of tested RBCs) × 100. The suppression effect within each group was calculated as follows using the formula provided by Enegide et al. [121]:

(1) $$Suppression\;witjin\;group\;\%=\frac{\;Parasitemia\;\left(pre\right)-Parasitemia\;\left(post\right)}{Parasitemia\;\left(pre\right)}$$

Histopathological examination of brain (cerebrum, cerebellum, and hippocampus) 

At the end of experiment, the mice groups were dissected, and the whole brain was immediately removed, then washed in 0.9% sodium chloride solution and placed instantly in 10% formalin. The tissues were dehydrated in ascending grades of ethyl alcohol, cleared in xylene, and finally embedded in paraffin wax blocks. The paraffin blocks were cut on glass slides using a rotary microtome at 4 μm thickness. The deparaffinized brain slices were thoroughly cleaned in 100 percent ethyl alcohol before being stained with Ehrlich’s hematoxylin [122]. A Philips digital pathology solutions microscope and imaging system (Phillips Intelli Site Ultra-Fast Scanner, FMT0225, Eindhoven, Netherlands) was used to investigate the brain sections at three regions (cerebrum, cerebellum, and hippocampus).

#### 4.3.2. Prophylactic Protocol (Protective Screening) 

In this experiment, twenty-four adult mice were split into four groups at random (n = 6).

Group 1: Normal NOT infected mice as control.

Group 2: Negative control; mice were administered distilled water followed by infection with 10^6^ *Plasmodium berghei*-infected RBCs.

Group 3: Mice were administered doses of 70 mg/kg/day of betalains followed by infection with 10^6^ *Plasmodium berghei*-infected RBCs.

Group 4: Positive mice received doses of 5 mg/kg/day of chloroquine followed by infection with 10^6^ *Plasmodium berghei*-infected RBCs.

The experimental mice primarily received distilled water, betalains, and chloroquine for 4 consecutive days. On the fifth day of the experiment, mice under experiment were inoculated with standard inoculums of 10^6^ *Plasmodium berghei*-infected RBCs with 21% parasitemia level. The parasitemia level was estimated regularly in all mice every two days post-infection (48, 96, 144 h). 

At the end of the experimental period (after 6 days of infection), blood samples were drawn from the orbital plexus using capillary tubes to assess the hematological parameters assay (CBC) and placed in EDTA tubes [123]. Additionally, all animal groups were sacrificed under mild anesthesia by diethyl ether, and the brain was excised, cleaned, and homogenized in PBS and stored at −20 °C until use for estimation of the antioxidant activities, inflammatory cytokines, and neurotransmitters. 

Enzyme-linked immunosorbent assay (ELISA) kits, using the DYNEX DSX best 20,000 Automated ELISA System from DYNEX Technologies, Chantilly, VA, USA), were used to conduct biochemical experiments. This microplate immunoanalyzer is fully automated and suited for medium-sized labs. Up to 48 tests can be conducted on 4 microplates at once in a single profile.

Determination of Antioxidant Markers (NO, SOD, and GSH) and MDA

Nitric oxide (NO) was determined using the Mouse Total Nitric Oxide ELISA kit (Cat. No. MBS720290); superoxide dismutase (SOD) activity was measured via the Mouse SOD ELISA kit (Cat. No. MBS034842); and the malondialdehyde (MDA) level was determined via the Mouse Malondialdehyde ELISA kit (Cat. No. MBS741034), which were purchased from MYBioSource. Glutathione (GSH) was assayed using an ELISA kit purchased from Elabscience, (Houston, TX, USA), (Cat. No. E-EL-0026). All procedures were performed according to the manufacturers’ protocols. 

Determination of Inflammatory Cytokines’ Markers 

The interleukins IL-6, IL-12, and IL-1β and tumor necrosis factor alpha (TNF-α) levels were determined using the Mouse IL-6 ELISA kit (Cat. No. ab100713), Mouse IL-12 ELISA kit (Cat. No. ab213866), Mouse IL-1beta ELISA kit (Cat. No. ab197742), and Mouse TNF-α ELISA kit (Cat. No. ab100747), respectively. All kits were purchased from Abcam Inc. Waltham, MA, USA. Meanwhile, IL-10 levels were estimated using the Mouse IL-10 ELISA kit (Cat. No. MBS018124), and Interferon-gamma (INF-γ) levels were estimated using the Mouse INF-γ ELISA kit (Cat. No. MBS2500105), which were acquired from MYBioSource, San Diego, CA, USA. All procedures were performed according to manufacturers’ protocols. 

Determination of Brian Neurotransmitters (Epinephrine, Norepinephrine, and Serotonin)

Norepinephrine (NE) levels were estimated using the Mouse Norepinephrine ELISA kit (Cat. No. MBS9346061); epinephrine levels were estimated via the Mouse Epinephrine ELISA kit (Cat. No. MBS162837); and serotonin/5hydroxytryptamine (5-HT) levels were estimated via the Mouse Serotonin ELISA kit (Cat. No. MBS723181); all kits were purchased from MYBioSource and all procedures were according to the manufacturers’ protocols. 

### 4.4. Statistical Analysis

GraphPad®️ Prism Statistical Package Version 9 (2022) was used to visualize the data. The Statistical Package for the Social Sciences for Windows (v. 21; IBM Corp., Armonk, NY, USA) was used to analyze the data. Data are expressed in terms of the mean and standard deviation (SD). Tests of normality (Shapiro–Wilk) were used for data distribution assessments. Parametric tests were performed on normally distributed data. One-way analysis of variance (ANOVA) was used to compare various groups, followed by the post hoc least significant difference (LSD) multiple comparison test. Significance was accepted at *p* < 0.05.

## 5. Conclusions

Based on our findings, betalains derived from *Beta vulgaris* succeeded in combating the parasitemia load induced by *P. berghei* in male mice, whether using it in a therapeutic or preventive capacity, at a concentration of 70 mg/kg. This was demonstrated through alleviating the histopathological, inflammatory, and oxidative stress, as well as the hematological changes caused by *Plasmodium* on the brain. Nevertheless, more research is needed to establish the exact means by which betalains can attenuate parasitemia and its complications induced by *Plasmodium* on the brain.

## Figures and Tables

**Table 1:** Percentages of parasitemia in *P. berghei*-infected mice on day 9 of the curative experiment.

Groups	Parasitemia %	
	Pre-Treatment	Post-Treatment
Group 1 (Negative control group)	35.40 ± 9.71	51.20 ± 12.27 ^a^*
Group 2 (Chloroquine 5 mg/kg)	44.20 ± 8.58	19.40 ± 12.62 ^a^**^b^****
Group 3 (Betalains 70 mg/kg)	41.20 ± 15.61	17.00 ± 10.41 ^a^**^b^****

Data are expressed as mean ± std. deviation (SD); n = 6; superscript ^a^ represents significance compared to pre-treatment between groups within the same raw. Significance within the same column by ^b^ represents significance compared to negative control post-treatment group; significance at * *p* < 0.05, ** *p* < 0.01, **** *p* < 0.0001.

**Table 2:** Suppression percentages of malaria infection on day 9 of the curative experiment.

Groups	Suppression %
Group 1 (Negative control group)	−47.46 ± 28.67
Group 2 (Chloroquine 5 mg/kg)	59.27 ± 22.64 ^a^****
Group 3 (Betalains 70 mg/kg)	61.72 ± 16.23 ^a^****

Data are expressed as mean ± std. deviation (SD); n = 6; superscript ^a^ represents significance compared to distilled water-treated group. Significant at **** *p* < 0.0001.

**Table 3:** Percentages of parasitemia and inhibition of *Plasmodium berghei*-infected mice in all mice every two days (2, 4, 6) in the prophylactic test.

Groups	Parasitemia and Inhibition%					
	Day 2	In%	Day 4	In%	Day 6	In%
Group 2 (Negative control group)	1.00 ± 0.81	-	26.00 ± 11.76	-	48.16 ± 8.37	-
Group 3 (Betalains 70 mg/kg)	0.45 ± 0.32	55	18.33 ± 9.20 ^a^*	29.50	24.16 ± 6.11 ^a^****	49.82
Group 4 (Chloroquine 5 mg/kg)	0.23 ± 0.15	77	9.00 ± 5.32 ^a^****^b^*	65.38	20.00 ± 4.47 ^a^****	58.47

Data are expressed as mean ± std. deviation (SD); n = 6. Significance within the same column by superscript ^a^ represents significance compared to negative control group. ^b^ represents compared to betalains 70% group. Significant at * *p* < 0.05, **** *p* < 0.0001. In% = inhibition percentage for each group compared to group 1 within the same day.

## Data Availability

Data are contained within the article.
